# Recent Advancements in the Research of the Mechanism by Which PPAR‐γ Is Involved in Postoperative Neurocognitive Disorder

**DOI:** 10.1096/fj.202502727R

**Published:** 2025-11-17

**Authors:** Yubing Huang, Yuhang Zhu, Zhaoqiong Zhu

**Affiliations:** ^1^ Department of Anesthesiology Affiliated Hospital of Zunyi Medical University Zunyi China; ^2^ Early Clinical Research Ward Affiliated Hospital of Zunyi Medical University Zunyi China

**Keywords:** central nervous system, inflammatory response, oxidative stress, perioperative neurocognitive disorders, peroxisome proliferator‐activated receptor γ

## Abstract

Perioperative neurocognitive disorders (PND) have emerged as prevalent central nervous system complications during the perioperative phase in contemporary medical practice. Despite their significance, the existing clinical diagnostic and therapeutic approaches for PND remain suboptimal, and the underlying pathogenesis continues to be a focal area of research within the realm of neurocognition. Accumulating evidence has indicated that peroxisome proliferator‐activated receptor γ (PPAR‐γ) assumes a pivotal role in the pathogenic mechanisms and regulatory processes of PND. The PPAR‐γ signaling pathway is intricately associated with multiple pathophysiological processes, including inflammatory responses, oxidative stress, β‐amyloid protein (Aβ) aggregation, and aberrant phosphorylation of Tau protein. This review article comprehensively examines the role of PPAR‐γ in the onset and progression of PND. By synthesizing current knowledge, it aims to provide a framework for further in‐depth investigations into PND‐related mechanisms centered around the PPAR‐γ pathway, thereby facilitating the development of novel therapeutic strategies and diagnostic tools.

## Graphical abstract

PPAR‐γ plays a crucial role in the pathogenic mechanisms and regulation of PND. Its pathways are intricately intertwined with inflammatory responses, oxidative stress, β‐amyloid deposition, and abnormal phosphorylation of Tau protein.

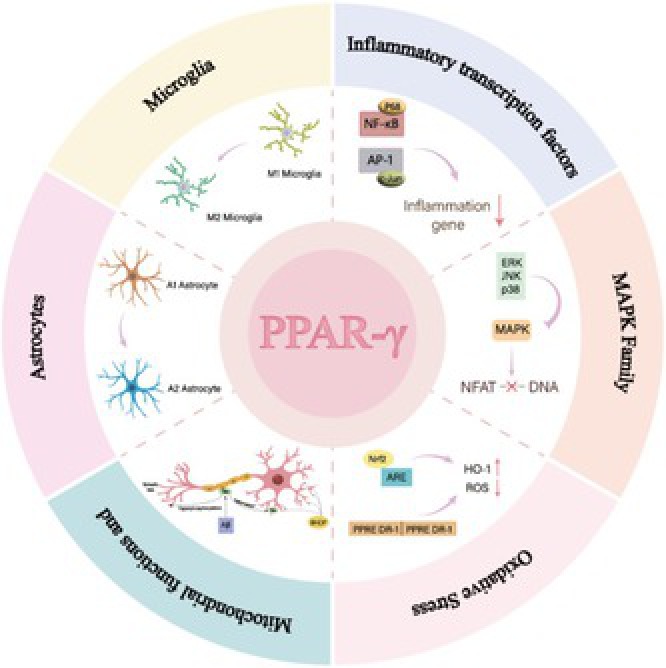

## 1 Introduction

With the accelerating trend of population aging, PND, a central nervous system (CNS) complication characterized by complex pathophysiological mechanisms and significant clinical consequences, has emerged as a critical and unresolved medical challenge. PND can manifest at diverse time points during the perioperative phase. It has the potential to impact postoperative cognitive functions such as memory, attention, and orientation. Consequently, this may lead to an elevated incidence of adverse reactions and complications, a deterioration in the quality of patients' recovery, an elongation of the average hospital stay, and even an increase in the mortality rate [1]. The Pathophysiological Mechanism of PND encompasses multiple aspects, including neuroinflammatory responses, oxidative stress, alterations in neurotransmitter levels, and abnormal neural network configurations. Among these, neuroinflammatory responses play a pivotal role in the onset and progression of PND. Numerous prior investigations have demonstrated that agonists of the PPAR‐γ can mitigate or even prevent neuroinflammatory responses, thereby decreasing the incidence of PND [2]. Peroxisome proliferator‐activated receptors (PPARs) belong to the nuclear hormone receptor superfamily. To date, three distinct PPAR subtypes, namely PPAR‐α, PPAR‐β, and PPAR‐γ, have been identified, and they exhibit structural homology. In recent years, the role of PPAR‐γ in neurodegenerative disorders has drawn increasing research focus. A variety of PPAR‐γ ligands have been proposed as potential therapeutic strategies for treating cognitive dysfunction [3]. Consequently, the action mechanism of PPAR‐γ in PND has emerged as a focal point in contemporary research on neurocognitive function. This review article meticulously examines the incidence and regulatory effects of PND. The exploration encompasses aspects such as the gene expression modulation of PPAR‐γ, the signaling cascades in which it is involved, and its functions within cells and tissues.

## 2 Comprehensive Analysis of the Association Between PND and the Neuroinflammatory Mechanism

**FIGURE 1:**
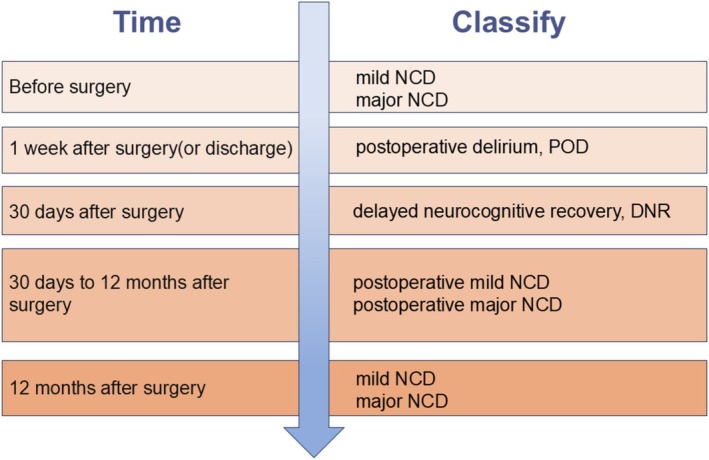
Classification of PND.

PND represents a prevalent and crucial complication during the perioperative phase. In elderly patients undergoing major or high‐risk surgical procedures, its incidence can reach up to 50% [4]. Based on the fifth edition of the Diagnostic and Statistical Manual of Mental Disorders (DSM‐5), PND is classified into five subcategories according to the time of onset, as depicted in Figure 1. The underlying mechanism of PND may be associated with neuroinflammatory responses, oxidative stress, alterations in neurotransmitter levels, and abnormalities in neural networks, among other factors [5].

Currently, within the realm of clinical practice, there exist no well‐defined strategies for the prevention, management, and treatment of PND. Given that the neuroinflammatory mechanism is most intricately associated with the onset of PND, suppressing the emergence of detrimental neuroinflammation might emerge as one of the pivotal approaches for the prevention and control of PND [6]. A meta‐analysis demonstrated that in orthopedic patients who received parecoxib following general anesthesia surgery, there was a notable reduction in the interleukin‐6 (IL‐6) level. This reduction was accompanied by a substantial decline in the incidence of postoperative cognitive impairment [7]. Additional investigations have demonstrated that ulinastatin is capable of effectively attenuating the systemic inflammatory response through the inhibition of neutrophils and monocytes. In elderly patients undergoing hip replacement surgery, the administration of ulinastatin can decrease the incidence of postoperative cognitive dysfunction [8]. Furthermore, in postoperative patients and those in the intensive care unit, the utilization of dexmedetomidine can mitigate the inflammatory response. This is achieved by reducing the activation of the signaling pathway, thereby lowering the incidence of PND [9]. Simultaneously, the perioperative application of dexmedetomidine can also curtail the release of inflammatory factors, alleviate postoperative stress, and downregulate the expression of tumor necrosis factor, consequently ameliorating the symptoms of PND [10]. Among various pharmacological agents, statins have been shown to exert pleiotropic effects beyond lipid‐lowering, including independent anti‐inflammatory and antioxidant properties, which contribute to the reduction of systemic inflammatory markers such as C‐reactive protein (CRP). In patients already on long‐term statin therapy (e.g., atorvastatin) prior to surgery, continuation of treatment throughout the perioperative period may confer protective benefits against the development of PND [2]. Nevertheless, the existing evidence is derived from studies with limited sample sizes, and future investigations with larger cohorts may yield more robust and statistically significant outcomes. The aforementioned clinical research findings comprehensively validate that the implementation of drug intervention strategies during the perioperative phase to impede the onset and progression of central nervous system inflammation can significantly mitigate the incidence of PND. This discovery holds substantial clinical practical implications. The research outcomes further suggest that an in‐depth exploration of neuroinflammation‐associated signaling cascades and the identification of pivotal regulatory targets therein possess crucial theoretical significance and practical viability for the establishment of a scientific prevention and control framework for PND. It is anticipated to offer novel perspectives and targets for the clinical formulation of more efficacious preventive and therapeutic strategies against PND.

In clinical perioperative investigations, it has been demonstrated that during the processes of anesthesia and surgical procedures, a substantial quantity of inflammatory mediators is generated. These mediators can trigger the activation of glial cells within the cerebral parenchyma, leading to an augmented production of pro‐inflammatory cytokines and an upregulation of the expression of central inflammatory markers. This cascade of events culminates in the dysfunction of neurons and synapses, ultimately resulting in postoperative neurocognitive impairment [11]. Accumulating evidence from several studies suggests that PND and Alzheimer's disease (AD) might share common neuropathological mechanisms [12]. In animal models of PND, it has been noted that the neuropathological alterations in mice following anesthesia and surgery bear resemblance to those observed in AD. These alterations encompass microglial hypertrophy and hyperplasia, astrocytic proliferation, Aβ deposition, and aberrant phosphorylation of the Tau protein [13]. Among the various factors involved in neuroinflammatory and neurodegenerative conditions associated with astrocytes and microglia, PPAR‐γ assumes a pivotal regulatory role. An elevation in its activity is conducive to mitigating the inflammatory response within the central nervous system and attenuating the severity of cognitive deficits [14]. Furthermore, pro‐inflammatory signaling cascades, including nuclear factor kappa B (NF‐κB) and activator protein 1 (AP‐1), which are implicated in the regulatory network of PPAR‐γ, can also mediate the anti‐inflammatory and neuroprotective functions of astrocytes [15]. A substantial number of cohort investigations have demonstrated that baseline cognitive impairment or dementia serves as a crucial independent risk factor for PND. Specifically, it continuously elevates the risk of PND by a factor of 2 to 5 [16]. A prospective cohort study has revealed that in elderly patients undergoing anesthesia and surgical procedures, the elevated concentrations of phosphorylated Tau proteins (Tau‐PT217 and Tau‐PT181) in preoperative plasma exhibit a high degree of correlation with the incidence and severity of PND [17]. The aforementioned evidence suggests that PND and the pathogenesis of AD share analogous pathophysiological mechanisms. The protective influence of PPAR‐γ on cognitive function following the regulation of neuroinflammation may offer novel insights into the prevention and management of PND and even AD. Consequently, exploring the regulatory mechanism of neuroinflammation centered around PPAR‐γ is conducive to formulating valuable reference recommendations for promoting clinical preventive and control strategies for PND.

## 3 Comprehensive Analysis of the Inflammatory Regulatory Function of Peroxisome PPAR‐γ

PPAR‐γ occupies a distinctive position, exhibits pleiotropic functions, and holds significant translational potential for disease treatment within the realm of inflammation regulation, particularly in the context of neuroscience. Initially, PPAR‐γ serves as a pivotal regulatory node in the intricate balance between anti‐inflammatory and pro‐inflammatory processes. Through antagonizing pro‐inflammatory transcription factors, including NF‐κB and AP‐1, PPAR‐γ effectively suppresses the expression of inflammatory cytokines such as tumor necrosis factor‐alpha (TNF‐α), IL‐6, and interleukin‐1 beta (IL‐1β). Secondly, PPAR‐γ has the capacity to upregulate anti‐inflammatory mediators like interleukin‐10 (IL‐10) and transforming growth factor‐beta (TGF‐β). This upregulation facilitates the transition of the inflammatory microenvironment from a “pro‐inflammatory” state to an “anti‐inflammatory” one, thereby contributing to the restoration of a more homeostatic milieu [18]. Concurrently, PPAR‐γ plays a crucial role in reducing the maturation and release of IL‐1β by inhibiting the activation of the NLR family pyrin domain‐containing 3 (NLRP3) inflammasome. This molecular mechanism holds significant importance in neurodegenerative disease models, such as AD and Parkinson's disease, where dysregulated inflammation is a prominent pathological feature [19]. PPAR‐γ is also capable of integrating the neuro‐immune‐metabolic network. Within microglia and astrocytes, PPAR‐γ exerts inhibitory effects on M1 polarization, promotes the M2 anti‐inflammatory phenotype, and diminishes the release of neurotoxic factors, including reactive oxygen species (ROS) and nitric oxide (NO) [20]. Furthermore, via the PPAR‐γ‐dependent antioxidant pathway, such as the synergistic interaction with nuclear factor erythroid 2‐related factor 2 (Nrf2), it can mitigate oxidative stress, facilitate the restoration of mitochondrial function, and safeguard neurons [21]. As a pivotal factor in metabolic regulation, PPAR‐γ can enhance insulin resistance and rectify lipid metabolism disorders, thereby disrupting the vicious cycle of “metabolic abnormalities‐chronic inflammation‐nerve injury” [22]. Clinically, PPAR‐γ receptor agonists, such as rosiglitazone and pioglitazone, were previously utilized for the treatment of diabetes. Currently, their notable neuroprotective effects in animal models have led to their repositioning as therapeutic agents for neuroinflammation [23]. PPAR‐γ not only assumes a central regulatory role in neuroinflammation but also serves as a bridge between basic mechanisms and clinical translation. It plays an irreplaceable role in alleviating the incidence of PND subsequent to the emergence of perioperative neuroinflammation.

### 3.1 The Architectural Configuration and Distinctive Features of PPAR‐γ

PPAR‐γ, a ligand‐activated transcription factor, serves as a crucial therapeutic target for a diverse range of neurodegenerative disorders. The gene sequence of PPAR‐γ is mapped to the 3p25 region on the short arm of human chromosome 3. It encompasses nine exons and spans a total length exceeding 100 kb [24]. Analogous to other type II nuclear receptors, the protein architecture of PPAR‐γ consists of six domains (designated as A–F) within four functional domains. These functional domains are respectively the ligand‐independent activation domain (termed the A/B domain), the DNA‐binding domain (abbreviated as DBD, corresponding to the C domain), the hinge region (the D domain), and the ligand‐dependent ligand‐binding domain (comprising the E/F domain and the AF‐2 region) [25]. Structurally, the ligand‐independent transcriptional activation domain (region A/B) is situated at the N‐terminus. This domain plays a crucial role in modulating the expression of target genes. The ligand‐binding domain is positioned at the C‐terminus. Upon binding to the ligand, it enables receptor dimerization. Functionally, within region A/B of PPAR‐γ, the activity can be impeded by the phosphorylation of the ser273 residue by mitogen‐activated protein kinases (MAPK). The AF‐2 region, which is encompassed within the ligand‐binding domain (LBD), has the ability to interact with recruited co‐factors. This interaction facilitates transcriptional activation.

The DNA‐binding domain (region C) exerts its function by specifically recognizing and binding to the peroxisome proliferator response element (PPRE) within the promoter region of the responsive gene, thereby regulating the expression of downstream target genes. Region D, also referred to as the hinge region, serves as a connector between the DNA‐binding domain and the ligand‐binding domain (region E/F). The binding of numerous nuclear factors to region D can have an impact on the activity of PPAR‐γ [26].

### 3.2 Modulation of PPAR‐γ Expression

PPAR‐γ encompasses three distinct subtypes, namely PPAR‐γ1, PPAR‐γ2, and PPAR‐γ3, as detailed in Table 1. Among these, PPAR‐γ1 exhibits a near‐universal expression pattern across almost all cell types. PPAR‐γ2 is predominantly expressed in white adipose tissue and brown adipose tissue. In contrast, PPAR‐γ3 is mainly detected in macrophages, adipose tissue, and the colon [27]. All subtypes of PPAR‐γ play pivotal roles in adipocyte differentiation and glucose metabolism. PPAR‐γ is extensively distributed within the brain tissue. Specifically, it is present in neurons, astrocytes, microglia, and oligodendrocytes, etc. Moreover, it demonstrates high levels of expression in the piriform cortex, dopaminergic neurons, basal ganglia, and reticular formation [28]. Upon activation, PPAR‐γ forms a heterodimeric complex with the nuclear receptor 9‐*cis* retinoic acid (RXR). This complex has the ability to bind to deoxyribonucleic acid (DNA) and interact with various cofactors, including steroid receptor coactivator 1 (SRC1), CREB‐binding protein (CBP)/p300, and peroxisome proliferator‐activated receptor gamma coactivator‐1α (PGC‐1α). Subsequently, it induces the transcriptional activation of numerous genes involved in metabolism, mitochondrial function regulation, inflammatory response modulation, and oxidative stress response [29]. Simultaneously, PPAR‐γ is a ligand‐activated nuclear receptor. Upon binding to its ligand, it initiates transcription for subsequent metabolic modulation. The ligands of PPAR‐γ can be classified into endogenous and exogenous types. Endogenous ligands encompass long‐chain fatty acids (LCFA), oxidized fatty acids like 9‐oxo‐12(Z)‐octadecenoic acid and 10‐oxo‐12(Z)‐octadecenoic acid, as well as hydroxy fatty acids such as hydroxyoctadecadienoic acid. These endogenous ligands play a crucial role in promoting adipocyte differentiation, maintaining the functionality of adipose tissue, and influencing disease progression by regulating apoptosis and survival signaling pathways in tumors and neurodegenerative disorders [30]. The exogenous ligands of PPAR‐γ are predominantly synthetic compounds, including medications for hypoglycemic and lipid‐lowering purposes, particularly thiazolidinedione drugs. The process of post‐translational modifications (PTMs) significantly impacts the activity and functional status of PPAR‐γ. Research has demonstrated that alterations in PTMs of PPAR‐γ, including phosphorylation, ubiquitination, acetylation and deacetylation, *O*‐GlcNAc glycosylation modification, and S‐nitrosylation, etc., directly affect its spatial configuration, regulate protein–protein interactions, and the affinity between the receptor and the ligand. Consequently, these modifications influence the transcription of downstream target genes of PPAR‐γ [31].

**TABLE 1:** A comparative analysis of the characteristics of the three subtypes of PPAR‐γ.

Characteristic	PPAR‐γ1	PPAR‐γ2	PPAR‐γ3
Protein structure	Lack of the N‐terminal extension of 30 amino acids specific to PPAR‐γ2	Absence of the N‐terminal extension of 30 amino acids that is unique to PPAR‐γ2	Lack of the N‐terminal extension of 30 amino acids specific to PPAR‐γ2
Tissue distribution	Widely expressed (the most prevalent)	Nearly restricted to adipose tissue	Adipose tissue, macrophages, and colonic epithelium
Principal functions	Anti‐inflammation, insulin sensitization, regulation of lipid metabolism	Lipogenesis, adipocyte differentiation, energy storage	Show a high degree of similarity to PPAR‐γ1
Significance of regulation	Mediate the anti‐inflammatory, neuroprotective, and cardiovascular protective effects of PPAR‐γ in non‐adipose tissues	The key regulators of metabolic functions in obesity and diabetes mellitus	Limited studies have been conducted on its functions. It may share similarities with the functions of PPAR‐γ1 or exhibit fine‐tuning functions under specific circumstances

PPAR‐γ exhibits its highest level of expression within adipocytes. It assumes a pivotal role in the modulation of adipogenesis, the maintenance of energy homeostasis, and the regulation of lipid biosynthesis [32]. In brown adipose tissue, PGC‐1α is extensively expressed. It occupies a central position in orchestrating the gene expression of crucial components involved in mitochondrial biogenesis. Moreover, PGC‐1α serves as a key molecular entity in regulating metabolic processes across numerous vital organs, encompassing both white and brown adipose tissues, skeletal muscle, the heart, the liver, and the kidney [33]. PGC‐1α has the ability to bind to PPAR‐γ and augment the transcriptional activity mediated by it. These two molecules collaborate to promote the expression of uncoupling protein 1 (UCP1) within mitochondria and to govern the oxidative metabolic capacity of mitochondria [34].

### 3.3 Peroxisome PPAR‐γ and the Nervous System

Bidirectional signaling between neurons and microglia is the basis for the normal functioning of the CNS. When microglia are continuously activated, it can trigger chronic neuroinflammatory responses, which can interfere with neuronal function and disrupt the signaling between neurons and microglia [35]. Among the three subtypes of PPARs, PPAR‐γ is a key neuronal subtype in the CNS. PPAR‐γ can regulate the expression of anti‐inflammatory cytokines and inhibit pro‐inflammatory signaling pathways, such as the TLR4/NF‐κB and JAK/STAT1 pathways, thereby suppressing the gene transcription and expression of pro‐inflammatory cytokines. Activated PPAR‐γ can transform microglia from a pro‐inflammatory state to an anti‐inflammatory state, playing an important neuroprotective role [36]. Immunohistochemical studies in rodents and humans have shown that PPAR‐γ is expressed in neurons and neuroglial cells in many important brain regions, including the prefrontal cortex, basal ganglia, thalamus, and hippocampus, indicating that PPAR‐γ plays an important role in maintaining CNS homeostasis [37].

Numerous investigations within the realm of neuroscience have demonstrated that PPAR‐γ exhibits a significant association with a multitude of neurodegenerative disorders. These include AD, Parkinson's disease (PD), amyotrophic lateral sclerosis (ALS), multiple sclerosis (MS), Huntington's disease, among others [38]. In the context of these neurodegenerative pathologies, PPAR‐γ exerts a protective function via multiple intricate mechanisms. These mechanisms encompass anti‐inflammatory responses, antioxidant activities, metabolic modulation, and anti‐apoptotic effects. Notwithstanding the formidable challenges encountered in the process of translating preclinical findings into clinical applications, the development of targeted therapeutic strategies directed against PPAR‐γ continues to be a research avenue with substantial promise in the field of neuroscience.

## 4 The Neurobiological Action Mechanism of PPAR‐γ in PND

**FIGURE 2:**
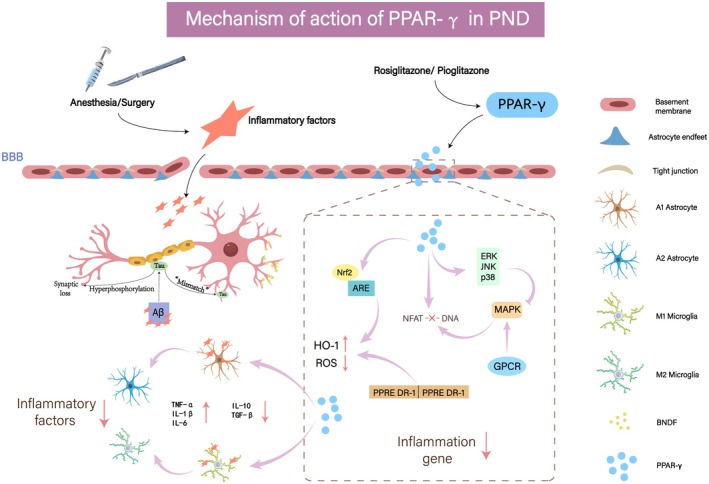
Mechanism of action of PPAR‐γ in PND.

In the context of PND, the action mechanism of PPAR‐γ encompasses multifaceted regulatory processes. As depicted in Figure 2, PPAR‐γ predominantly confers a protective effect via multiple intricate mechanisms. These include anti‐inflammatory responses, antioxidant activities, safeguarding the integrity of the blood–brain barrier (BBB), facilitating synaptic plasticity, and modulating metabolic pathways.

### 4.1 The Molecular Mechanism Underlying Gene Expression Regulation and the Associated Signaling Cascades Implicated in PND With a Focus on the Role of PPAR‐γ

#### 4.1.1 PPAR‐γ and Inflammatory Transcription Factors

**FIGURE 3:**
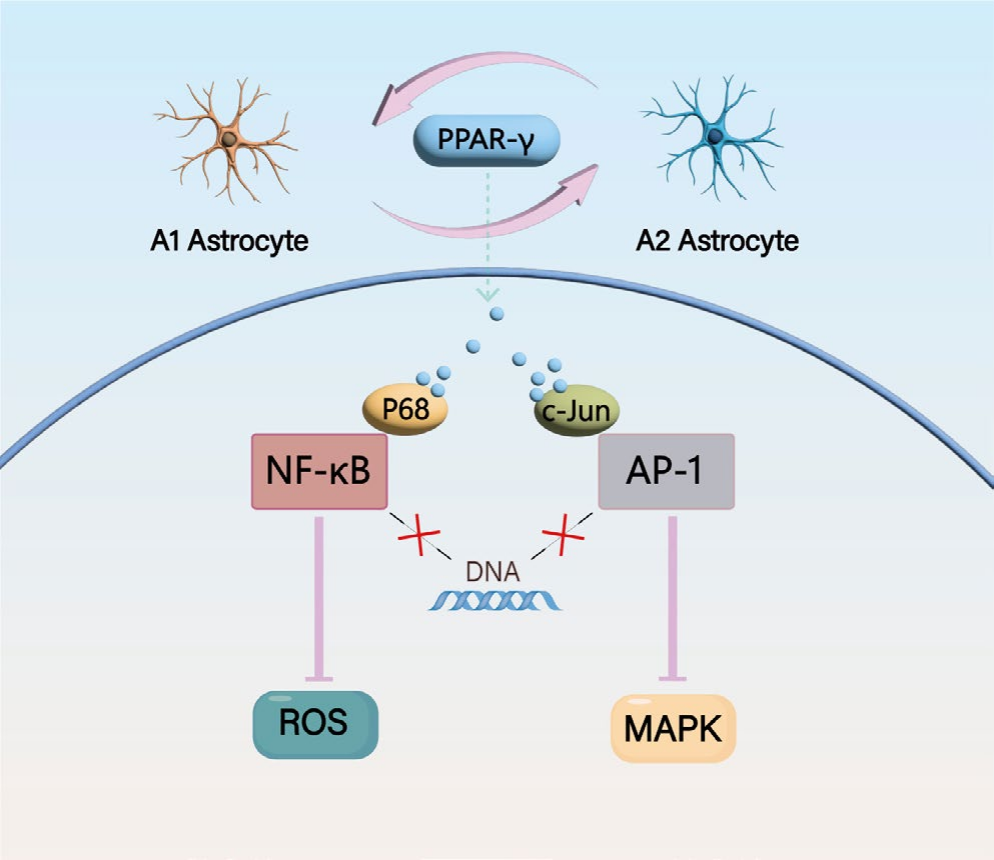
The interplay between the NF‐κB/AP‐1 pathway and PPAR‐γ.

Inflammatory response and oxidative stress are pivotal mechanisms underlying the onset of PND. PPAR‐γ also assumes a critical role in this pathological process. PPAR‐γ has the capacity to impede the signal transduction of NF‐κB. NF‐κB serves as the core transcription factor in the inflammatory response cascade. Specifically, PPAR‐γ binds to the p65 subunit of NF‐κB, thereby obstructing its DNA‐binding ability [39]. Simultaneously, PPAR‐γ competitively occupies co‐activators (such as CBP/p300), effectively suppressing NF‐κB‐mediated gene transcription [40]. Furthermore, by inhibiting the activity of NF‐κB, PPAR‐γ significantly downregulates the expression of TNF‐α, IL‐6, inducible nitric oxide synthase (iNOS), and intercellular adhesion molecule‐1 (ICAM‐1). This, in turn, leads to a reduction in the generation of ROS and leukocyte infiltration, contributing to the attenuation of the inflammatory and oxidative stress responses associated with PND [41]. PPAR‐γ has the capacity to interfere with the binding of the transcription factor AP‐1 to DNA, thereby diminishing AP‐1‐mediated transcriptional activity of inflammatory genes [42]. AP‐1, a transcription factor complex consisting of c‐Jun, c‐Fos, among others, plays a crucial role in regulating genes associated with inflammation and cell proliferation [43]. PPAR‐γ interacts directly with the c‐Jun subunit of AP‐1 or recruits corepressors (e.g., NCoR/SMRT) to disrupt the binding of AP‐1 to the DNA promoter region (such as the TRE sequence). Additionally, it downregulates the MAPK pathway, including JNK and ERK. This downregulation leads to a reduction in the N‐terminal phosphorylation of c‐Jun (at Ser63/73 residues), subsequently attenuating the transcriptional activity of AP‐1 [44]. As a result, the expression of AP‐1‐dependent inflammatory genes, such as matrix metalloproteinase‐9 (MMP‐9) and cyclooxygenase‐2 (COX‐2), is inhibited, ultimately alleviating tissue damage [45], as shown in Figure 3.

#### 4.1.2 PPAR‐γ and the MAPK Family

The MAPK family, which includes ERK, c‐Jun N‐terminal kinase (JNK), and p38, serves as a pivotal signaling hub in inflammatory and stress responses. Upon activation, PPAR‐γ induces the expression of MAPK phosphatase (MKP‐1). MKP‐1 inactivates ERK, JNK, and p38 through dephosphorylation, effectively blocking their downstream signal transduction cascades [46]. In the upstream kinase cascade reaction, PPAR‐γ suppresses the activity of Raf kinase within the Ras–Raf–MEK–ERK signaling pathway or impedes the activation of JNK/p38 by apoptosis signal‐regulating kinase 1 (ASK1) [47]. The inhibition of the MAPK pathway can mitigate the accumulation of ROS, including superoxide anion and hydrogen peroxide, and downregulate the expression of pro‐apoptotic proteins such as Bax and Caspase‐3, which is conducive to cell viability [48]. In the G protein‐coupled receptor (GPCR)‐Gαq/11‐MAPK‐nuclear factor of activated T cells (NFAT) pathway, GPCRs can initiate MAPK signal transduction via the Gαq/11 subunit. This activation stimulates phospholipase C (PLC), leading to the generation of inositol trisphosphate (IP3) and diacylglycerol (DAG), and promotes the nuclear translocation of NFAT, subsequently activating protein kinase C (PKC) and MAPK [49]. PPAR‐γ competes with NFAT for binding to co‐activators (e.g., CREB‐binding protein, CBP) or directly occupies the promoter regions of NFAT target genes (such as interleukin‐2, IL‐2, and interleukin‐4, IL‐4), thereby inhibiting their transcriptional activity. By inhibiting the calcium release mediated by the IP3 receptor, PPAR‐γ reduces the dephosphorylation of NFAT by calcineurin, preventing NFAT from translocating into the nucleus. Through blocking NFAT and MAPK signals, PPAR‐γ decreases the expression of chemokines (such as C‐C motif chemokine ligand 2, CCL2, and C‐X‐C motif chemokine ligand 8, CXCL8) and adhesion molecules (such as vascular cell adhesion molecule 1, VCAM‐1), thus restricting the migration of inflammatory cells [50].

#### 4.1.3 PPAR‐γ and Oxidative Stress

Nrf2, a pivotal transcription factor, plays a crucial role in modulating the antioxidant response and influencing the development of oxidative stress. It establishes a positive feedback loop with the expression of PPAR‐γ, and is intricately involved in concurrently regulating the expression of transcription factors and their target antioxidant genes [51]. Investigations have revealed that Nrf2 can directly bind to the antioxidant response elements (ARE) within the 784/764 and 916 regions of the PPAR‐γ promoter region [52]. In vivo research demonstrated that the expression of PPAR‐γ was significantly diminished in Nrf2 gene knockout mice, further substantiating the direct regulatory role of Nrf2 over PPAR‐γ [53]. Furthermore, the PPAR‐γ agonist 5‐hydroxy‐4‐phenyl‐fumaramate (5H4PB) is capable of activating the Nrf2/ARE signaling cascade. This activation leads to the upregulation of ARE‐dependent cytoprotective genes, including heme oxygenase‐1 (HO‐1), catalase, and superoxide dismutase (SOD). Such upregulation is of paramount importance in the cellular defense mechanism against oxidative stress [54]. Simultaneously, the activation of PPAR‐γ can modulate the expression of endothelial nitric oxide synthase (eNOS) and iNOS. Research findings have indicated that the aortic segments of endothelial‐specific PPAR‐γ knockout mice exhibit reduced NO production. This suggests that PPAR‐γ safeguards cells from oxidative stress by regulating the expression of eNOS [55]. HO‐1 serves as a pivotal antioxidant enzyme and is also a gene under the regulatory control of Nrf2. The activation of HO‐1 has the potential to mitigate cellular oxidative stress, dampen the inflammatory response, and decrease the apoptosis rate. The peroxisome proliferator response element direct repeat‐1 (PPRE DR‐1) functions as the response element of PPAR‐γ. Within human vascular cells, PPAR‐γ elicits the expression of HO‐1 through its interaction with two PPRE DR‐1 elements positioned between 1740 and 1826 kb of the transcriptional start site [56]. The cluster of differentiation 36 (CD36) receptor, a scavenger receptor, is capable of recognizing and internalizing oxidized lipids and can be regulated by PPAR‐γ as well [57]. Research investigations have shown that treatment with PPAR‐γ ligands can upregulate the expression of CD36 in murine macrophages [58].

### 4.2 The Molecular Mechanism by Which PPAR‐γ Modulates Mitochondrial Function and Synaptic Plasticity in the Context of Neuroscience Research

Brain‐derived neurotrophic factor (BDNF) and long‐term potentiation (LTP) play pivotal roles in cognitive processes, memory consolidation, and learning mechanisms. In the critical processes of learning and memory formation, mitochondria are intricately involved in the LTP of synaptic transmission, which is fundamental for information encoding and retrieval in the neural network. PGC1‐α, acting as a PPAR‐γ enhancer, serves as a key transcriptional co‐activator in mitochondrial biogenesis and cellular energy metabolism pathways. Notably, it exhibits high expression levels in the brain, underscoring its significance in neural function. Autopsy analyses of AD patients have revealed a significant decline in the expression of PGC1‐α in the hippocampus [59]. This reduction in PGC1‐α expression is associated with a decrease in mitochondrial density across multiple brain regions, concomitantly leading to a decline in adenosine triphosphate (ATP) levels [60]. Synaptic functionality is closely dependent on dendritic spines, and the loss of dendritic spines is directly correlated with the impairment of synaptic function. BDNF is recognized as one of the key mediators in enhancing dendritic density and promoting synaptic plasticity [61]. Accumulating research has demonstrated that PPAR‐γ agonists exert a beneficial impact on enhancing synaptic plasticity. For instance, rosiglitazone has been shown to prevent the atrophy of dendritic spines and improve synaptic function [62]. Similarly, in rats administered with Aβ and treated with pioglitazone, a reduction in caspase‐3 activation and an elevation in BDNF levels were observed, suggesting an improvement in synaptic plasticity [63].

Analogous to AD, the aggregation of Aβ and the hyperphosphorylation of Tau protein are pivotal pathological mechanisms underlying the onset of PND. Soluble Aβ and hyperphosphorylated Tau protein serve as crucial initiators of synaptic dysregulation [64]. Early investigations have demonstrated that the neurodegenerative cascade culminating in AD is instigated by Aβ. This peptide can trigger aberrant processing of the amyloid precursor protein (APP). PPAR‐γ exerts an inhibitory effect on the processing of endogenous APP to generate Aβ via modulation of β‐secretase 1 (BACE1) and γ‐secretase activities. Consequently, this action reduces Aβ deposition [65]. Under physiological conditions, the expression of Tau protein is predominantly restricted to axons. Nevertheless, numerous studies have revealed that an elevation in the level of Aβ can reallocate Tau protein to dendrites and dendritic spines via a “mismatch” mechanism. This process is concomitantly accompanied by the hyperphosphorylation of Tau protein, which subsequently triggers synaptic loss [66]. Accumulating evidence has indicated that upon the activation of PPAR‐γ, the levels of Thr181, Ser396, and Ser416 residues, which are implicated in the phosphorylation of Tau protein within the cerebral cortex of mice, are all downregulated [67]. Furthermore, several scholars have reported that following the administration of a PPAR‐γ receptor agonist, the pathological alterations in hippocampal neurons and the phosphorylation of Tau protein in AD model mice are effectively inhibited. This intervention is conducive to enhancing the memory function of AD‐like mice. This phenomenon might be associated with the involvement of the G protein‐coupled acetylcholine receptor signaling pathway and the protein kinase B/glycogen synthase kinase 3β (GSK3β) axis in the pathophysiology of AD [68].

Although the Tau and Aβ pathologies associated with AD may elevate the risk of PND or be implicated in its pathophysiological process, the utilization of animal models to investigate their roles in PND is still beset with limitations. Firstly, numerous transgenic mouse models of AD, such as APP/PS1 and 5xFAD, can mimic Aβ deposition. Nevertheless, they typically lack significant neuronal loss, which is one of the core hallmarks of human AD. This deficiency renders these models inadequate in providing sufficient evidence for studying long‐term cognitive impairments associated with PND, thereby highlighting the disparities in pathological manifestations attributable to species differences. Secondly, there are also constraints in model construction. The majority of AD mouse models simulate Aβ pathology by transgenic overexpression of genes associated with familial AD (FAD) mutations or mimic Tau pathology by overexpressing mutant MAPT genes. However, the vast majority of human PND cases are sporadic, and there is no such overexpression of these genes. This artificial overexpression may not accurately mirror the latent and subtle Aβ or Tau pathological alterations in PND [69]. Furthermore, PND is often precipitated by a combination of multiple factors, including surgical trauma, anesthesia, pain, and inflammation. Surgery and anesthesia themselves can induce neuroinflammation and disrupt the blood–brain barrier. These effects may be independent of or not entirely reliant on Aβ/Tau pathologies but can interact with them. Models solely featuring Aβ or Tau pathologies may not comprehensively capture these intricate interactions [70]. Finally, other co‐pathologies, such as α‐synucleinopathy, are frequently present in the brains of human AD and PND patients. Additionally, systemic factors, such as Aβ/Tau accumulation in peripheral organs, systemic inflammation, blood–brain barrier dysfunction, and sleep rhythm disturbances, are challenging to fully replicate in standard mouse models. Nevertheless, these factors may play a role in the onset and progression of PND [71]. In conclusion, although Tau and Aβ pathologies may be involved in the development of PND, studying their roles in PND using existing animal models confronts multiple challenges, encompassing species differences, limitations in model construction, the difficulty of simulating acute multiple perioperative insults, and the absence of co‐pathologies.

### 4.3 The Regulatory Mechanism of PPAR‐γ in Astrocytes and Microglia Cells

Surgical trauma and pain can trigger the release of systemic inflammatory mediators. These mediators penetrate the CNS via the BBB, thereby activating astrocytes and microglia, which ultimately results in central neuroinflammation [72]. Astrocytes, the most prevalent cell type within the brain parenchyma, play multifaceted roles. They are responsible for regulating neurotransmitter and calcium homeostasis. Additionally, they are involved in crucial processes such as the formation, maturation, and pruning of synapses. Moreover, astrocytes are essential for maintaining the integrity and function of the blood–brain barrier, controlling ion balance and cerebral blood flow, and providing metabolic and trophic support to the neurons in the brain [73]. In the context of neuroinflammation, astrocytes serve as significant regulators. They can exacerbate the inflammatory response by recruiting peripheral immune cells, activating CNS‐resident microglia, and through their own intrinsic neurotoxic mechanisms [74]. In neurodegenerative disorders or CNS injuries, astrocytes undergo hyper‐activation, a phenomenon known as reactive astrogliosis. During this state, they secrete pro‐inflammatory cytokines such as TNF‐α, IL‐6, IL‐1β, and NO. Notably, the activation of PPAR‐γ can counteract the pro‐inflammatory actions of astrocytes [75]. Reactive astrocytes can be classified into two distinct states: A1 and A2. The A1 state exhibits a detrimental, pro‐inflammatory phenotype, while the A2 state is associated with neuroprotective and reparative functions [76]. The PPAR‐γ agonist telmisartan facilitates the phenotypic transition of A2 astrocytes and inhibits the transformation of A1 astrocytes through the suppression of the NF‐κB signaling pathway. Simultaneously, PPAR‐γ downregulates the expression of inflammatory mediators by counteracting pro‐inflammatory signaling cascades such as NF‐κB and AP‐1, thereby mitigating neuroinflammation [77]. Furthermore, activated PPAR‐γ can upregulate antioxidant enzymes within astrocytes, including superoxide dismutase (SOD) and glutathione peroxidase (GSH‐Px), among others. This action reduces the accumulation of ROS, safeguarding neurons from oxidative insult [78]. In the context of maintaining the BBB, the end‐feet of astrocytes encircle cerebral blood vessels and play a crucial role in preserving the integrity of the BBB. Activated PPAR‐γ restricts the infiltration of peripheral inflammatory cells and toxic substances into the brain parenchyma by enhancing the expression of tight junction proteins and decreasing the permeability of the BBB [79].

Microglia, a subset of glial cells within the CNS, serve as resident macrophages in the brain parenchyma [80]. Anesthetic exposure and surgical procedures can elicit varying degrees of microglial activation, thereby initiating an inflammatory cascade. Surgical interventions may also induce degranulation of CNS mast cells, potentially triggering microglial activation and subsequent neuronal injury, ultimately contributing to the development of PND [81]. Neuroinflammatory processes and microglial activation are implicated in the pathogenesis of PND. Analogous to macrophages, activated microglia can be phenotypically classified into M1, characterized by pro‐inflammatory and neurotoxic properties, and M2, which exhibit anti‐inflammatory and neuroprotective functions [82]. The phenotypic shift of microglia from M2 to M1 is pivotal in microglial activation and the ensuing neuroinflammatory responses. Activation of PPAR‐γ exerts inhibitory effects on M1 microglia. This leads to a reduction in the release of pro‐inflammatory cytokines, including TNF‐α, IL‐1β, and IL‐6. Moreover, it can induce the polarization of microglia towards the M2 phenotype, thereby promoting the secretion of anti‐inflammatory factors such as IL‐10 and TGF‐β [83]. Analogous to astrocytes, PPAR‐γ can also upregulate antioxidant enzymes, such as SOD and GSH‐Px, within microglia. This action alleviates the oxidative stress experienced by microglia. Simultaneously, it inhibits the activity of nicotinamide adenine dinucleotide phosphate (NADPH) oxidase, thereby reducing the production of ROS [84].

As can be deduced from the foregoing discussion, the activation of PPAR‐γ has demonstrated notable potential in the realm of neuroprotection. Telmisartan, as previously mentioned, stands apart from other classic agonists such as pioglitazone owing to its distinctive multi‐modal mechanisms of action. As an angiotensin II receptor blocker (ARB) medication, telmisartan functions as a selective PPAR‐γ modulator. This property enables it to confer benefits such as blood pressure reduction, enhancement of insulin sensitivity, and anti‐inflammatory effects. Simultaneously, it circumvents the common side effects associated with traditional thiazolidinediones (TZDs) drugs, including weight gain and fluid retention (edema secondary to sodium and water retention) [85]. Consequently, for patients concurrently presenting with hypertension and metabolic disorders, telmisartan emerges as a superior therapeutic option.

### 4.4 Additional Molecular Mechanisms Implicated in PPAR‐γ

The interplay between gut microbiota dysbiosis and PND remains a focal point in contemporary clinical neuroscience research. Investigations have revealed that a decline in beneficial bacterial species, such as *Lactobacillus* and *Bifidobacterium*, during the perioperative phase elevates the susceptibility to postoperative cognitive deficits. Moreover, surgical anesthesia further exacerbates the dysregulation of the gut microbiota composition [86]. Accumulating evidence indicates a bidirectional connection between the gut microbiota and the CNS. This connection enables the gut microbiota to exert influence on brain function and behavior via neural, endocrine, and immune‐mediated pathways [87]. Short‐chain fatty acids (SCFAs), key bioactive metabolites of the gut microbiota, serve as crucial signaling molecules within the microbiota‐gut‐brain axis (MGB). They play a pivotal role in modulating various mental and neurological disorders [88]. Notably, SCFAs possess the ability to traverse the BBB. As such, microbiota‐derived SCFAs can transmit information from the gut to the brain through the MGB, thereby fine‐tuning brain function and potentially influencing cognitive outcomes [89]. In the most recent investigation within the realm of neuroscience, it has been discovered that acetyl‐CoA synthetase 2 (ACSS2) functions as a crucial target for eliciting rapid and enduring antidepressant responses. Specifically, upon the binding of PPAR‐γ to ACSS2, a molecular cascade is initiated. This interaction activates the transcription process of tryptophan hydroxylase 2 (TPH2), thereby regulating the antidepressant‐like effects mediated by SCFAs via the ACSS2‐TPH2 axis [90]. These research findings offer additional and compelling evidence regarding the involvement of PPAR‐γ in modulating central nervous cognitive functions, specifically through the mediation of the MGB. This work contributes significantly to the growing body of knowledge in the field of neuroscience, further elucidating the complex molecular mechanisms underlying antidepressant actions and central nervous system regulation.

Insulin signaling plays a pivotal role in safeguarding the survival and maintaining the homeostasis of neurons. Moreover, it is also involved, to a certain degree, in the intricate processes of learning and memory [91]. Aberrant regulation of insulin signaling compromises neuronal oxidative metabolism [92]. In a particular investigation, upon treating high‐fat diet (HFD) rats with a PPAR‐γ activator, it was observed that neuronal insulin resistance was alleviated. Concurrently, the generation of mitochondrial ROS, alterations in mitochondrial membrane potential, and mitochondrial swelling within the brain were mitigated. These findings suggest that the activation of PPAR‐γ can counteract HFD‐induced neuronal insulin resistance and brain mitochondrial dysfunction, thereby enhancing cognitive function [93]. Furthermore, non‐pharmacological strategies and lifestyle interventions are integral components of the regulatory mechanism of PPAR‐γ [94]. It has been documented that lifestyle interventions can augment hippocampal neurogenesis and learning capabilities in aged rodents. The underlying mechanism is associated with the release of neurotrophic factors by neurons during synaptic activity [95].

## 5 Concluding Remarks

PPAR‐γ is a pivotal transcription factor that assumes a critical role in the pathophysiology of PND. PPAR‐γ is implicated in multiple facets associated with PND, including the inflammatory response mechanism, oxidative stress response, Aβ aggregation, aberrant phosphorylation of Tau protein, and gut microbiota dysregulation. The modulation of PPAR‐γ activity is intricately intertwined with numerous key pathway regulatory mechanisms, thereby exerting a far‐reaching influence on the overall neurocognitive system. Contemporary research has verified that the administration of PPAR‐γ agonists during the perioperative period facilitates the amelioration of central nervous system injury induced by anesthesia and surgery in humans, consequently enhancing neurocognitive function. A multitude of PPAR‐γ ligands have been proposed as potential therapeutic agents for cognitive impairment. Notwithstanding, owing to the inconsistent outcomes of clinical trials and the lack of tissue selectivity of numerous PPAR‐γ agonists, which may trigger the activation of PPAR‐γ in peripheral tissues, the utilization of these ligands as therapeutic drugs remains encumbered by numerous limitations. The pathophysiological mechanism of PND is intricate. Although PPAR‐γ has the ability to suppress inflammatory cascades such as the NF‐κB pathway, perioperative stress might exacerbate cognitive deficits via alternative pathways. Consequently, the efficacy of single‐target intervention remains restricted. Notwithstanding the significant advancements achieved in the research of PPAR‐γ within the cardiovascular system, immune system, lipid metabolism, and anti‐tumor mechanisms, further investigations are still warranted in the domain of CNS cognitive function. PND encompasses a multitude of processes, including neuroinflammation, neurotransmitter dysregulation, and epigenetic modulation. Future research endeavors will focus on elucidating the intricate mechanisms by which PPAR‐γ interacts with other pivotal pathways, such as the NLRP3 inflammasome and the gut–brain axis. Moreover, investigations will delve into the alterations in the PPAR‐γ signaling pathway across diverse age groups and in the context of comorbid conditions, such as diabetes mellitus. These areas of inquiry represent crucial directions for advancing our understanding of PND and developing targeted therapeutic interventions. Hence, additional research focusing on PPAR‐γ as a therapeutic target holds promise for uncovering novel strategies for the prevention and treatment of central nervous system disorders.

## Author Contributions

All authors contributed to the study conception and design. Y.H. wrote the first draft and created figures. Z.Z. and Y.Z. revised the manuscript. All authors edited and approved the final manuscript.

## Conflicts of Interest

The authors declare no conflicts of interest.

## Data Availability

The authors have nothing to report.
